# Single-cell genomics: coming of age

**DOI:** 10.1186/s13059-016-0960-x

**Published:** 2016-05-10

**Authors:** Sten Linnarsson, Sarah A. Teichmann

**Affiliations:** Department of Medical Biochemistry and Biophysics, Karolinska Institutet, Unit of Molecular Neurobiology, Scheeles väg 1, 171 77 Stockholm, Sweden; Wellcome Trust Sanger Institute, Wellcome Genome Campus, Hinxton, CB10 1SD UK

Single-cell genomics is the study of the individuality of cells using omics approaches. Although young, the field has now entered its teenage years and is beginning to show clear signs of maturity. Its origins can be traced back to pioneering experiments that allowed the detection of gene expression in single cells by microarrays (reviewed in [1]). However, it was with the emergence of “next-generation” DNA sequencing that single-cell genomics really took off [2–4]. Although initial experiments were modest in size and resulted in noisy and incomplete data, they immediately revealed the great potential for biological discoveries. It soon became clear that the substantial technical and biological variability required data from many single cells in order to allow meaningful data mining and interpretation of the data [5]. Thus, the following years were spent pursuing a few lines of development: improvements in the accuracy and scope of single-cell methods and increasing throughput and reducing cost. Today, we are in a position to routinely measure gene expression in tens of thousands of single cells with high accuracy in terms of quantification of gene expression (albeit sensitivity in terms of detection of mRNAs varies significantly depending on protocol and sequencing depth). The costs are at least manageable and continue to decrease.

While single-cell RNA-seq is now mature and almost routine, technological development has shifted to other modalities: DNA, protein, chromatin modifications, and more. Single-cell whole-genome DNA sequencing is challenging because loss of material causes dropouts in the sequence and because sequencing errors are difficult to distinguish from real mutations. Despite these challenges, single human cortical neurons have been used to reconstruct lineages based on somatic mutations that had accumulated during development [6]. Similarly, clonal evolution within solid tumors can be revealed by detecting somatic copy number variations in single cells (reviewed in [7]).

Another trend is the extension of single-cell analysis to measure epigenetic states such as DNA accessibility [8–10], methylation [11], and chromosome conformation [12]. Generally these methods pose similar challenges to DNA sequencing but offer access to pure cellular epigenetic states that are simply inaccessible by bulk methods.

Single-cell protein analysis occupies a different niche, where smaller numbers of proteins can be analyzed but in very large numbers of cells, classically using fluorescence-activated cell sorting (FACS) for up to eight targets but more recently with mass cytometry targeting up to hundreds of proteins [13]. A limiting factor for protein analysis remains the requirement for high-quality affinity reagents such as antibodies.

Finally, a recent development (but see [14]) is the combination of methods to simultaneously measure two or more modalities in single cells. For example, genome and transcriptome [15, 16], transcriptome and methylome [17, 18], and RNA and protein [19]. In the near future, such experiments will be able to link the phenotypes of single cells evolving in tumors to their genotypes.

Due to the speed with which single-cell genomics technologies are evolving, computational analysis methods are racing to keep up. Statistical and computational methods are at the heart of single-cell genomics and are critical to extracting meaningful information and biology from the data. Much work has focused on transcriptomic data analysis (e.g., reviewed in [20]) and in this special issue of *Genome Biology* there are examples of areas that benefit from bespoke computational approaches at the levels of both cells and genes. In terms of individual genes, a method to define significant differences in the cell-to-cell variation in gene expression (as opposed to mean expression levels) is reported [21] and one paper addresses expression states of long noncoding RNAs [22]. In terms of cell-to-cell variation at the DNA level, there is clearly tremendous scope for computational method innovation in the area of tumor heterogeneity, addressed by Beerenwinkel and colleagues [23], and Markowetz and Ross [24] in this issue.

## Recent applications

Single-cell RNA sequencing has had a profound impact on our understanding of neuronal and hematopoietic cell types, as well as the immune system. Examples of novel insights in immunity include a window on to an unexpected plethora of dendritic cells in mouse immunity [25] and new regulators and subpopulations of CD^4+^ T cells [26–28]. In hematopoiesis, much single-cell transcriptomics work has focused on hematopoetic stem cells and the single-cell perspective has provided resolution of proliferation phenotypes [29–31]. A broader view of early specification of hematopoietic cell types was recently provided by Paul et al. [32]. Mead and colleagues [33] provide new insights into the erythroid–myeloid decision in this special issue.

While these publications all focus on mouse as a model, the unbiased nature of single-cell RNA sequencing provides great discovery potential in less-well-studied animals. An example of this is the profiling of platelets (thrombocytes) from hematopoietic stem cells in zebrafish by Macaulay et al. [34]. In this issue, Pearson and Molinaro profile single cells in planarian regeneration [35]. Looking to the future, this type of approach can be expanded to comparative studies of many organisms across the animal kingdom in order to gain insight into the evolution of cell types.

The applicability of single-cell transcriptomics to nonadherent cells, such as those of hematopoiesis and immunity, is perhaps not surprising: these cells naturally exist as individual cells and remain stable after single-cell capture by FACS or in microfluidic devices. In the area of neurobiology and neuronal cell populations, the success of single-cell RNA sequencing is more surprising as these cells are bound up within networks of adherent junctions. Recently, comprehensive maps of cell types and subtypes have been produced for a number of key brain regions, including developing and adult cerebral cortex, and the day will come when we will have a full catalog of molecularly defined cell types in the whole nervous system. A particularly appealing application of such a reference atlas is in the use of human cerebral organoids to model human brain (which is otherwise inaccessible) in development and disease [36]. The fact that novel cell states, cell populations, and factors have been validated in this domain bodes well for a broader remit of single-cell transcriptomics to solid organs and tissues.

The DNA dimension, i.e., tracking mutations, copy number variations, and chromosomal aberrations at the single-cell level, has been important in both somatic cell populations such as neurons, as well as in cancer. In this issue, Park and colleagues show how single-cell dissection of tumor heterogeneity can translate directly into new combinatorial therapies in a xenograft model [37].

## Future prospects

Gazing into our crystal ball, it is easy to predict an ever-increasing role for single-cell genomics in discovery science, translational applications, and even ecology. The major driver of the single-cell genomics revolution is the step change in resolution of DNA and epigenetic and RNA sequencing down to the level of an individual cell. Since the cell is the basic building block of an organism, sequencing each cell in isolation provides information that is fundamentally different from genomic data that relates to ensembles of cells.

In terms of single-cell transcriptomics, the RNA content of a cell is deeply informative about its phenotype and function. This technique is so powerful and informative that it is likely that the community will ultimately map all mammalian organs, tissues, and cell types at single-cell resolution. A comprehensive resource such as this, effectively a “human cell atlas”, would be a tremendously useful and unique reference data set for biology and medicine.

Like many previous waves of biotechnology, single-cell genomics started in academia and basic research but is now set to move into pharma and the clinic. Once an atlas of human cell types is available, any diseased tissue can be compared with it. Cancer, in particular, the prototypical single-cell disease, will be particularly apt for a single-cell analysis overhaul. Diagnostic assays, which are currently based on crude bulk methods, will be tremendously more powerful once they are brought down to the level of the individual transformed cell, in the context of its surrounding tissue, with cell-type specificity and a full understanding of somatic mutations.

We are excited to be part of a community that has already achieved a lot, as showcased in this special issue, yet clearly still has a long and interesting journey ahead of it.

## Abbreviation

FACS: fluorescence-activated cell sorting
